# Twelve‐month change in quantitative MRI calf muscle fat fraction in CMT1A predicts clinical change over 4 years

**DOI:** 10.1002/acn3.52314

**Published:** 2025-02-17

**Authors:** Matthew R. B. Evans, Hamza A. Salhab, Christopher D. J. Sinclair, Sachit Shah, Michael G. Hanna, Tarek A. Yousry, John S. Thornton, Jasper M. Morrow, Mary M. Reilly

**Affiliations:** ^1^ Queen Square Centre for Neuromuscular Diseases National Hospital for Neurology and Neurosurgery London UK; ^2^ Department of Neuromuscular Diseases Queen Square UCL Institute of Neurology London UK; ^3^ Lysholm Department of Radiology National Hospital for Neurology and Neurosurgery London UK; ^4^ Neuroradiological Academic Unit Queen Square UCL Institute of Neurology London UK

## Abstract

**Objective:**

We measured clinical and quantitative MRI outcome measures in CMT1A to assess long‐term responsiveness, establish longitudinal validity and assess MRI as a bridging biomarker.

**Methods:**

Twenty patients with CMT1A and 20 matched controls underwent MRI, myometry and clinical assessments up to four times over mean 4‐year follow‐up. Bilateral calf muscle MRI included T1‐weighted sequences with Mercuri grading and three‐point Dixon quantitative fat fraction assessment. Patients were grouped on baseline calf muscle fat fraction: normal <5%, intermediate 5%–70% and end stage >70%.

**Results:**

Controls showed no significant change on MRI. CMT1A patients' calf muscle fat percentage progressed across all follow‐up visits: mean absolute change was +1.3 ± 1.2% (mean ± SD) at 12 months, +2.3 ± 2.2% at 27 months and 2.8 ± 2.9% at 49 months. Mercuri grades increased by 0.07 ± 0.11 per year. Responsiveness of individual muscle fat was less than for both calves combined. Patients with intermediate baseline calf muscle fat showed greater progression of 3.7 ± 2.3% at 27 months. There was strong correlation between rate of progression of calf muscle fat and CMT Examination Score (*ρ* = 0.71, *P* = 0.005). Calf muscle fat progression at 12 months correlated significantly with annualised CMT Examination Score progression at final visit (*ρ* = 0.65, *P* = 0.01).

**Interpretation:**

We demonstrated a consistent progression of calf muscle MRI fat over 4 years, significant longitudinal correlation between CMT Examination Score and calf muscle fat, and potential as a bridging biomarker by 1 year change in fat correlating with long‐term clinical progression. Increasing study duration minimally increased responsiveness; however, selecting patients with intermediate fat fraction significantly increased responsiveness.

## Introduction

Charcot Marie Tooth disease (CMT) is the eponymous term for the hereditary motor and sensory neuropathies which cause progressive disability due to distal wasting, weakness and sensory loss. CMT1A is the commonest subtype, comprising about half of all CMT patients, and has a prevalence of 1 in 5000.^1^ Advances in the understanding of the genetic mechanisms in CMT have led to the identification of potential drug treatments which require assessment for efficacy in clinical trials.^2^ Clinical outcome measures such as the CMT Neuropathy Score progress very slowly^3^ meaning clinical trials with large numbers of patients or long duration would be required which may not be practical in rare diseases. This has led to the conclusion that biomarkers which predict meaningful progression in functional and patient reported outcome assessments are needed to allow CMT clinical trials of 2 years or less.^4^

Lower limb muscle fat quantification with MRI has been investigated at a potential outcome measure in many inherited neuromuscular diseases,^5^ including several studies in patients with CMT. Cross‐sectional studies using T1‐weighted MRI sequences, which qualitatively assess muscle atrophy and fat accumulation, have demonstrated a distal gradient of involvement with only foot muscle involvement in the mildest CMT1A patients such as children and adolescents^6^ confirmed recently with muscle fat MRI quantification.^7^ In patients with moderate disease severity, there is a distal gradient within the calf muscles,^6^, ^8^, ^9^, ^10^ and a relative predilection for the anterolateral compartment and also medial head of gastrocnemius.^6^, ^8^, ^9^, ^10^, ^11^ These studies have also been useful at demonstrating cross‐sectional criterion validity through correlation with muscle strength, function and clinical scales such as the CMT Examination Score (CMTES).^8^, ^9^, ^10^, ^12^, ^13^, ^14^, ^15^

MRI quantification of muscle fat accumulation has also been shown to be responsive over 12 months in CMT1A,^12^, ^15^, ^16^, ^17^ as well as other forms of CMT and related disorders.^18^, ^19^ In particular, we previously published 12‐month follow‐up in 17 CMT1A patients, which demonstrated that bilateral calf muscle fat fraction was the most responsive outcome measure, with mean increase of 1.2%ff (SD 1.5%ff) with resultant large responsiveness, expressed as the standardised response mean (SRM), of 0.83.^12^ However, significant change in the clinical measures was not observed due to the short duration of these studies. Consequently, correlation between change in clinical measures and MRI measures was not seen, and the longitudinal validity of these outcome measures remains to be demonstrated. We have shown that responsiveness can be increased by including only patients with intermediate levels of muscle fat accumulation at baseline^15^ but whether this is the best method to increase responsiveness and how to practically implement this in a clinical are major unanswered questions.^4^

The current study presented is an extension of our CMT1A cohort's quantitative MRI, clinical and functional outcome measurements across 4 years with the aim of assessing long‐term responsiveness, establishing longitudinal validity and critically to correlate 1‐year quantitative MRI progression with long‐term clinical progression to assess utility as a bridging biomarker. We also present qualitative and quantitative analysis of individual calf muscles, to consider the practical optimisation of lower limb muscle MRI outcome measures in clinical trials of CMT1A.

## Methods

### Study design and participants

This study was an extension of the prospective longitudinal observational study^12^ of patients attending the inherited neuropathy clinics at the Queen Square Centre for Neuromuscular Diseases at the National Hospital for Neurology and Neurosurgery, London, United Kingdom. Inclusion criteria were genetic confirmation of chromosome 17p11.2 duplication for patients with CMT1A, and being aged 17 years or older. Exclusion criteria were concomitant diseases and MRI‐related contraindications. Healthy participants were enrolled as controls, matched for age, sex, weight, and body mass index distribution. All clinical and myometry assessments were done at the Queen Square Centre for Neuromuscular Diseases, University College London Institute of Neurology, United Kingdom. MRI was done at the National Hospital for Neurology and Neurosurgery, London, United Kingdom. Cross‐sectional and 12‐month quantitative MRI data have been reported previously.^12^ The study was approved by the local ethics committee and all participants provided written informed consent at enrolment.

### Procedures

At baseline, 20 patients with CMT1A and 20 matched controls were enrolled. All participants were invited to attend for four assessments spaced over up to 5 years. Each assessment comprised collection of demographic and clinical information on a standardised form, MRI, myometry and clinical evaluation as previously reported.^12^ Briefly, all CMT1A patients had the following assessments: medical history, neurological examination, bedside strength examination using MRC grading, SF‐36 quality of life questionnaire and the Charcot–Marie–Tooth examination score (version 2; CMTES). Patients and controls underwent detailed lower limb myometry on a HUMAC NORM dynamometer (CSMi, MA, USA). Participants were examined lying feet‐first and supine in a 3‐T MRI scanner (TIM Trio, Siemens, Erlangen, Germany). The quantitative lower limb muscle MRI protocol included axial acquisitions of both calves (lower legs) including 3‐point‐Dixon fat‐fraction quantification resulting in fat‐fraction maps expressed as percentage fat (0%–100%). T2‐weighed and magnetisation transfer ratio sequences were also collected at the additional time points and analysed but did not provide longitudinal data additionally useful to the Dixon sequence, so are not reported here. In addition at each scan, previously unreported T1‐weighted sequences (TR/TE = 671/16 ms, 10 slices, 10 mm thickness, 10 mm slice gap, 256 × 192 matrix, iPat acceleration of 2, 444 Hz/pixel bandwidth, TSE factor = 3, refocusing flip angle (fa) 130°, NEX = 2) and short‐tau inversion recovery sequences (TR/TE/Inversion Time = 5500/56/220 ms, NEX = 1, flip angle 180°, parallel imaging factor (iPat) = 2, 10 × 10 mm slices, 10 mm gap, 256 × 128 matrix (thigh), 256 × 128 matrix (calf)) were performed. The total acquisition time per participant per visit was about 35 min.

### Statistical analysis

**Figure 1:**
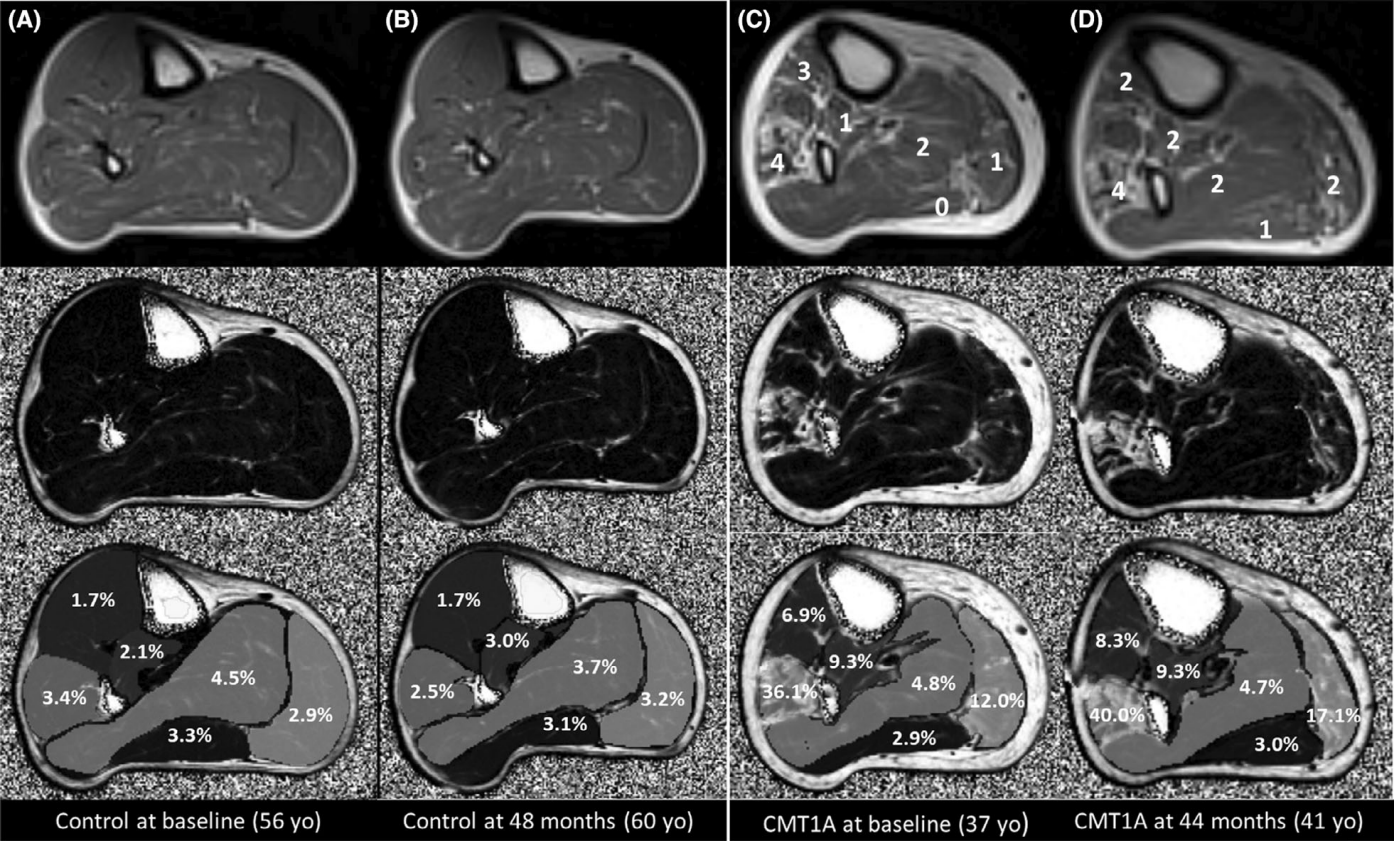
Example images. Axial MRI images of the right calf in a control at baseline (A) and last follow‐up (B), and patient at baseline (C) and last follow‐up (D). Top row: T1‐weighted images with modified Mercuri grades for the patient superimposed (based on whole muscle volume, all control muscles scored 0 on blinded analysis). Middle and bottom rows: Dixon Fat fraction maps (grayscale: 0%–100%ff) with muscle regions of interest and calculated mean fat fractions superimposed on bottom row. The control subject has normal fat fraction at baseline and does not significantly change over 4 years. The CMT1A patient shows variable fat fraction, highest in peroneus longus which increases in three muscles over 44‐month follow‐up.

A single observer (MRBE), different to the observer who performed the original analysis,^12^ manually performed all muscle segmentation for all scans, blinded to clinical details and visit number. Regions of interest (ROI) for each participant were drawn with reference to each other, to ensure equivalent ROI placement. Whole muscle regions of interest were defined bilaterally on the selected slice a fixed distance from the knee joint^12^ on an unprocessed Dixon acquisition (TE = 3.45 ms) using ITK‐SNAP software.^20^ ROI included the entire cross‐sectional area of the muscle up to but not including the surrounding fascia, and excluding prominent neurovascular structures. Left and right calf ROIs were thus defined for six muscles/muscle groups: tibialis anterior, peroneus longus, lateral gastrocnemius, medial gastrocnemius, soleus, and the posterior tibial group of muscles (Fig. 1). All maps were inspected visually for artefact. Muscles which had areas of gross artefact were excluded from the analysis. ROIs were transferred to the inherently co‐registered FF maps. Mean FF and cross‐sectional area (CSA) were extracted from the whole muscle regions of interest. All extracted data were checked for errors and outliers. In addition to the individual values, summary measures were calculated for the whole calf by CSA‐weighted average of FF from each relevant ROI.

As a measure of the contractile CSA, remaining muscle area (RMA) was defined for all measures as:

$$\mathrm{RMA}\;\left({\mathrm{mm}}^{2}\right)\;=\;\left({\mathrm{mm}}^{2}\right)\;\times \;\left(\left(100-\mathrm{fat}\;\text{fractio}\left(\%\right)\right)/100\right).$$

Longitudinal changes were quantified for each individual muscle and summary measures. Given the non‐uniformity of timings of visits three and four, longitudinal change in all parameters were converted to the mean follow‐up interval for that visit: 12 months for visit two, 27 months for visit three and 49 months for visit four.

For the qualitative analysis, the first and last scan for each participant were scored using a modified Mercuri scale, atrophy scale and scales of STIR hyperintensity and extent as described by Vivekanandam et al.^21^ The original Mercuri scale^22^ was converted to a 6‐point ordinal scale: 0—normal appearance; 1—early fat accumulation with scattered areas of T1 high signal; 2—numerous discrete areas of T1 high signal with beginning confluence <30% of the total muscle volume; 3—fat accumulation 30%–60% of the muscle volume; 4—fat accumulation >60% of the muscle volume; 5—end stage, no residual muscle tissue. Scoring was performed by consensus by two radiology consultants (SS and TAY) experienced in neuromuscular imaging, blinded to disease status and scan order.

All data were analysed using IBM‐SPSS Statistics Version 26.0. (Armonk, NY; IBM Corp.). All data were assessed for normality by the Shapiro–Wilk test alongside visual inspection of frequency distribution graphs. Responsiveness was evaluated for all outcome measures using the standardised response mean. SRM was categorised by magnitude according to Cohen's rule of thumb: <0.2, minimal responsiveness; 0.2–0.5, small responsiveness; 0.5–0.8, moderate responsiveness; >0.8, large responsiveness.

Summary statistics were calculated (mean ± standard deviation), for CMT1A and control groups, of each quantitative measure on an individual muscle, and summary measure basis. Mean baseline measures and mean change in individual muscle and summary measures were compared within groups by paired *t*‐test or Wilcoxon Rank Sum test, and between groups using independent two‐tailed *t*‐test or Mann–Whitney *U*‐test as appropriate. Inter‐muscle differences were assessed using ANOVA with post hoc comparisons using Bonferroni's method. Correlations were assessed using Pearson or Spearman correlation coefficients as appropriate. Patients were grouped based on baseline FF: normal <5%, intermediate 5%–70% and end stage >70% and rate of change in FF compared according to these categories. These cut‐offs were based on fat fraction values in controls, and those associated with end‐stage Mercuri grade. Statistical significance was set at *P* < 0.05.

## Results

As previously reported^12^ between January 2010 and July 2011, 20 patients with CMT1A (11 male, mean age 42.8y, standard deviation 13.9) and 20 healthy controls were recruited. Age, sex, height and weight were similar between the groups. At the visit two (mean 12.4 ± 1.0 SD; range 10.9–14.7 months), there remained 17 CMT1A patients and 20 controls. At visit three (27.4 ± 2.2; 23.5–30.9), there remained 14 CMT1A patients and 11 controls, and at visit four (47.2 ± 7.1; 34.9–56.8 months), there remained 10 CMT1A patients and 8 controls. Participant dropout for CMT1A patients was attributable to death, surgery, illness, inability to travel and personal reasons to withdraw from the study. For controls, withdrawal of their patient ‘buddy’ often meant that they also withdrew from the study.

### Baseline data

**Figure 2:**
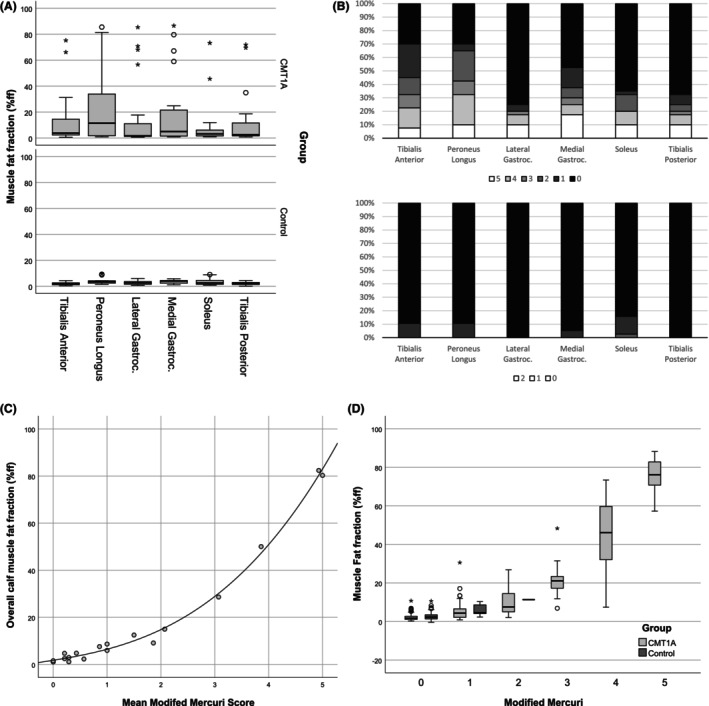
Baseline MRI. (A) Box plot of quantitative muscle fat fraction in lower limb muscles in CMT1A patients and controls. (B) Distribution of Mercuri grades in calf muscles in CMT1A patients and controls. (C) Scatter plot of mean muscle modified Mercuri grade and overall calf muscle fat fraction in CMT1A patients, fit line drawn as visual aid only. (D) Box plot of muscle fat fraction measured according to the muscle modified Mercuri grade. o = minor outlier, * = major outlier.

Example images are in Figure 1. Considering individual calf muscles in CMT1A patients at visit one, peroneus longus was the most affected followed by tibialis anterior and medial gastrocnemius, while soleus was the least affected for both quantitative fat fraction (Fig. 2A) and qualitative analysis with the modified Mercuri score (Fig. 2B). There was strong correlation between Mercuri grades and quantitative muscle fat fraction at an overall subject (*ρ* = 0.95, *P* < 0.001, Fig. 2C) and individual muscle level (*ρ* = 0.68, *P* < 0.001, Fig. 2D). Patients with a mean Mercuri grade of less than 0.5 generally had muscle fat fraction levels less than 5% that is similar to controls. STIR hyperintensity within calf muscles was rare with marked STIR hyperintensity noted in 1% of CMT1A patients' muscles (0% in controls) and mild STIR hyperintensity in 9% of CMT1A patients' muscles (6% of controls).

### Longitudinal data

Longitudinal quantitative MRI and selected clinical data are summarised in Table 1. As previously reported, there were no significant changes in any clinical measures in patients at 12 months (visit two). However, there was significant mean change in CMTES at visit three (mean 27 months) and visit four (mean 49 months), equivalent to 0.6 points per year of follow‐up. There was a similar significant change in CMTES‐lower limb components (CMTES‐LL) at both visits three and four. No other clinical or functional measure changed significantly over the study duration including no significant reduction in any lower limb myometry (data not shown). There were no significant changes in any of these measures in the control group over the study duration.

**Table 1:** Longitudinal data for bilateral calf muscle fat fraction.

	Baseline	12 months			27 months			49 months		
	Mean	Mean change	*P*	SRM	Mean change	*P*	SRM	Mean change	*P*	SRM
Calf muscle mean fat fraction (%ff)										
Controls	3.24 ± 1.60 (19)	0.20 ± 0.49 (19)	0.06	n.a.	0.02 ± 0.95 (10)	0.94	n.a.	0.43 ± 1.00 (6)	0.35	n.a
CMT1A all	16.16 ± 25.16 (20)*	1.28 ± 1.23 (17)	0.001	1.05	2.27 ± 2.15 (14)	0.001	1.05	2.76 ± 2.92 (9)	0.01	0.95
Baseline FF <5%	2.33 ± 1.41 (10)	0.29 ± 0.32 (8)	0.04	0.91	0.60 ± 0.44 (6)	0.02	1.37	0.83 ± 0.75 (4)	n.a.	n.a.
Baseline FF 5%–70%	17.16 ± 15.10 (8)	2.00 ± 1.13 (7)	0.004	1.71	3.71 ± 2.26 (6)	0.009	1.74	5.47 ± 3.3 (3)	n.a.	n.a
Baseline FF >70%	81.33 ± 1.49 (2)	2.72 ± 0.31 (2)	n.a	n.a.	2.96 ± 1.88 (2)	n.a	n.a.	2.55 ± 2.74 (2)	n.a.	n.a
Calf muscle cross‐sectional area (cm^2^)										
Controls	117.4 ± 27.3 (19)	1.1 ± 5.4 (19)	0.36	n.a	0 ± 3.9 (10)	0.99	n.a.	−2.2 ± 4.7 (6)	0.29	n.a
CMT1A	92.8 ± 24 (20)**	0.4 ± 3.8 (17)	0.63	n.a.	−4.7 ± 6.6 (14)	0.02	−0.71	−3.6 ± 6.1 (9)	0.13	n.a
Calf muscle remaining muscle area (cm^2^)										
Controls	113.6 ± 26.1 (19)	0.8 ± 5.2 (19)	0.48	n.a.	0 ± 4.1 (10)	0.99	n.a.	−2.5 ± 4.9 (6)	0.26	n.a.
CMT1A	79.7 ± 33.7 (20)**	−0.5 ± 4 (17)	0.59	n.a.	−5.3 ± 6.1 (14)	0.006	−0.87	−4.9 ± 6.5 (9)	0.06	n.a.
Clinical measures (CMT1A patients only)										
MRC Sum Score	207.8 ± 21.8 (20)	−0.1 ± 7 (17)	0.94	n.a.	−0.3 ± 6.4 (13)	0.99	n.a.	−2.3 ± 6.0 (9)	0.41	n.a.
SF36 total	73.9 ± 15.2 (18)	−2.7 ± 16.2 (16)	0.51	n.a.	−2.1 ± 14.7 (13)	0.59	n.a.	3.7 ± 7.2 (8)	0.16	n.a.
SF36 PF	64.7 ± 23.2 (18)	−1.2 ± 12.8 (16)	0.77	n.a.	−4.3 ± 8.3 (13)	0.09	n.a.	0.4 ± 15.7 (8)	0.83	n.a.
CMTES	8.0 ± 5.1 (20)	0.4 ± 1.3 (17)	0.37	n.a.	1.5 ± 1.5 (14)	<0.001	1.02	2.5 ± 1.6 (9)	<0.001	1.55
CMTES‐LL	6.3 ± 4.3 (20)	0.5 ± 1.2 (17)	0.12	n.a.	1.3 ± 1.1 (14)	<0.001	1.16	2.1 ± 1.7 (9)	<0.001	1.27

Data are presented as mean ± SD (*n*) and standardised to mean follow‐up intervals of 12, 27 and 49 months. *Patient baseline different from controls *P* < 0.05, **Patient baseline different from controls *P* < 0.01 *P*: paired *t*‐test. SRM given when change is significant.

n.a, not applicable; SRM, standardised response mean.

**Figure 3:**
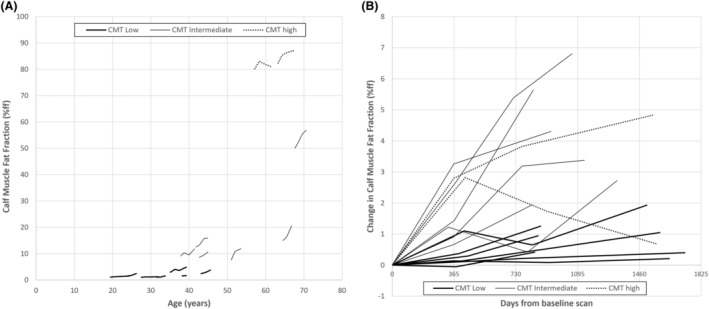
Calf muscle fat fraction MRI progression. Each line represents a single participant through the course of the study, stratified by baseline calf muscle fat fraction. (A) Scatter plot of calf muscle fat fraction by age in CMT1A patients. (B) Scatter plots of change in calf muscle fat fraction from baseline. The patients with low baseline fat fraction increase slowly over time, while those with intermediate fat fraction increase more rapidly. Those with end‐stage baseline fat fraction remain high with variability in measurement evident.

Controls showed no significant change from baseline calf muscle fat fraction, cross‐sectional area or remaining muscle area at any time point. By contrast in CMT1A patients, quantitative MRI measurement of calf muscle fat fraction continued to progress across all follow‐up visits (Table 1, Fig. 3). Mean change in calf muscle fat fraction was +1.28 ± 1.23%ff (mean ± SD) at 12 months, +2.27 ± 2.15%ff at 27 months and 2.76 ± 2.92%ff at 49 months. These cannot be directly compared due to patient drop‐out; however, individual patient trajectories are shown in Figure 3. These show that progression generally continues at the same rate for a given subject—that is there are slow progressors and fast progressors. For this reason, increasing follow‐up duration did not increase the standardised response mean (Table 1, Fig. 3B). Note that this relates to patients' disease progression in the calf muscles only, we have shown that more mildly affected children with CMT1A show progression in foot muscles,^7^ while in more severe forms of CMT such as CMT2A, progression is seen in the thigh muscles.^19^ There was significant progression of muscle atrophy in patients at visit three, whether measured by total cross‐sectional area or remaining muscle area. This lost significance at visit four, with fewer patients remaining.

Significant progression in intramuscular fat accumulation was also seen in qualitative grading of the T1w scans using the modified Mercuri grade. The mean calf muscle modified Mercuri score at baseline was 1.48 ± 1.74, while at final visit, mean Mercuri was 1.65 ± 1.81 (paired *t*‐test, *P* = 0.05). This translated in patients to a mean change in Mercuri grade of 0.07 ± 0.11 per year in patients, while the Mecuri grades in controls grades did not change significantly with mean change 0.02 ± 0.07 per year (*P* = n.s.). There was no significant longitudinal change in other qualitative MRI scores (atrophy, STIR) in patients or controls.

Responsiveness of individual muscle fat fraction was overall less than that for both calves combined (Table 2). Of the 12 individual calf muscles, seven showed significant change (*P* < 0.05) at visit 2 with SRM ranging from 0.45 to 0.78. Ongoing progression was seen across visits three and four, with overall progression at visit four ranging between 1.2 and 6.9%ff. There was at least a trend (*P* < 0.10) for all individual muscle progression at all visits. SRM were generally lower than the observed SRM for the overall calf fat fraction.

**Table 2:** Progression in muscle fat fraction by individual muscles.

Muscle	12 months				27 months				49 months			
	Change	SD	*P*	SRM	Change	SD	*P*	SRM	Change	SD	*P*	SRM
R. tibialis anterior	1.30	1.77	0.007	0.73	1.80	1.37	<0.001	1.32	3.35	3.53	0.029	0.95
L. tibialis anterior	0.87	1.78	0.055	0.49	1.34	1.81	0.012	0.74	3.69	4.23	0.027	0.87
R. peroneus longus	2.09	2.81	0.007	0.74	3.71	4.88	0.015	0.76	6.86	9.38	0.055	0.73
L. peroneus longus	1.85	3.63	0.051	0.51	2.03	3.41	0.056	0.60	3.10	4.77	0.072	0.65
R. lateral gastrocnemius	1.40	2.51	0.034	0.56	1.97	2.35	0.006	0.84	2.40	3.79	0.065	0.63
L. lateral gastrocnemius	0.92	1.33	0.017	0.69	1.79	2.31	0.011	0.78	3.78	5.71	0.075	0.66
R. medial gastrocnemius	1.45	2.49	0.030	0.58	3.04	3.78	0.008	0.80	4.69	7.27	0.072	0.65
L. medial gastrocnemius	1.45	2.99	0.053	0.49	4.28	7.66	0.056	0.56	6.44	8.14	0.035	0.79
R. soleus	0.80	1.02	0.005	0.78	1.16	1.12	0.001	1.03	1.19	2.04	0.096	0.58
L. soleus	1.18	2.61	0.073	0.45	2.52	5.41	0.088	0.47	4.83	7.89	0.055	0.61
R. tibialis posterior	1.33	2.59	0.050	0.51	2.42	3.09	0.011	0.78	2.90	3.14	0.027	0.92
L. tibialis posterior	2.13	4.40	0.058	0.48	2.48	3.90	0.026	0.63	5.41	7.33	0.028	0.74

*P*: significance of paired *t*‐test in patients compared with baseline, dark grey: significant changes, light grey: trend to significance.

L, left; R, right; SRM, standardised response mean.

### Baseline predictors of calf muscle fat fraction progression

Calf muscle fat fraction progression varies based on baseline fat fraction with patients with intermediate fat fraction at baseline showing greatest progression (Table 1, Fig. 3B). For example, at 27 months in the normal baseline group, calf muscle fat fraction had increased by 0.6 ± 0.4%ff (*P* = 0.02) while in the intermediate group, it had increased but 3.7 ± 2.3%ff (*P* = 0.009) (Table 1). However, due to change in the subgroups being more homogeneous, responsiveness was greater in both subgroups than in the whole cohort together (27‐month SRM normal baseline muscle fat fraction = 1.37, SRM intermediate baseline muscle fat fraction 1.74, SRM all CMT patients 1.05). There were only two patients with end‐stage calf muscle appearance (muscle fat fraction >70%) so limited conclusions could be drawn (dotted lines in Fig. 3). Although their muscle fat fraction remained high, there appeared to be more variability in measurement without consistent progression.

**Figure 4:**
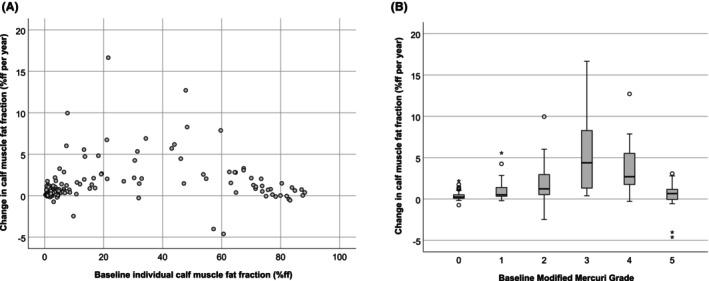
Individual calf muscle fat fraction MRI at baseline predicts subsequent progression. (A) Scatter plot of calf muscle fat fraction annualised progression by baseline value, each point represents a single muscle from a patient. (B) Box plot of calf muscle fat fraction progression by baseline modified Mercuri grade in that muscle. o = minor outlier, * = major outlier.

A similar relationship was seen if each individual calf muscle was considered separately (Fig. 4). Greatest rate of progression was seen in muscles with intermediate quantitative fat fraction (Fig. 4A) or intermediate Mercuri grades in that muscle at baseline (Fig. 4B). There were a limited number of STIR hyperintense muscles at baseline (*n* = 27/198). These increased by 1.60 ± 1.86%ff per year versus 1.04 ± 2.30%ff for muscles with normal STIR intensity (*P* = 0.09).

Given the importance of baseline calf muscle fat fraction in subsequent fat fraction progression, we assessed correlation between clinical measurements and calf muscle fat fraction across all time points. There were strong highly significant correlations between calf muscle fat fraction and CMTES (Spearman *ρ* = 0.798, *P* < 0.001) and between calf muscle fat fraction and the lower limb motor components of the CMTES (Spearman *ρ* = 0.813, *P* < 0.001). In terms of determining the cut‐off value of 5% fat fraction, receiver operating characteristic at area under curve of 0.857 and 0.849, respectively. Eighty percent of patient visits with CMTES scores less than 5 had calf muscle FF values <5%, while 86% of patient visits with CMTES scores >7 had calf muscle FF values >5%.

### Longitudinal correlation between clinical and MRI measures

**Figure 5:**
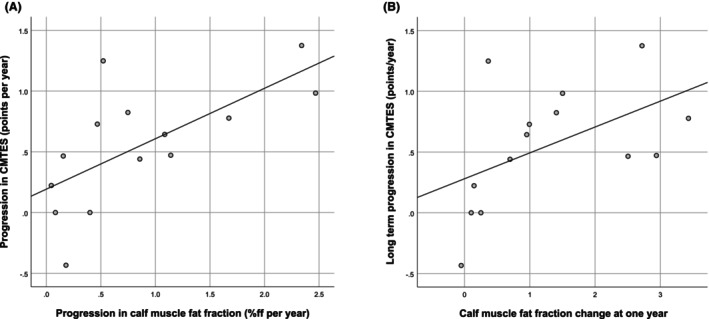
Longitudinal MRI‐clinical correlations in CMT1A patients with at least three visits. (A) Scatter plot of calf muscle fat fraction annualised progression at last follow‐up against CMTES annualised progression at last follow‐up (*ρ* = 0.71, *P* = 0.005). (B) Scatter plot of calf muscle fat fraction progression at 1 year against long‐term progression in CMTES (*ρ* = 0.65, *P* = 0.01).

We have previously demonstrated strong cross‐sectional correlation between calf muscle fat fraction and clinical measures including age, disease duration, CMTES and lower limb myometry^12^; however, the lack of change in clinical measures at 12 months meant we failed to show correlation in longitudinal change over this time period. The longer follow‐up in this study allowed us to see significant change in CMTES and CMTES‐LL. Considering all patients with at least three visits (*n* = 14), there was a strong correlation between rate of progression of calf muscle fat fraction and CMTES (*ρ* = 0.71, *P* = 0.005, Fig. 5A) and CMTES‐LL (*ρ* = 0.70, *P* = 0.006) at final visit. Calf muscle fat fraction progression at 12 months also correlated significantly with annualised CMTES (*ρ* = 0.65, *P* = 0.01, Fig. 5B) and CMTES‐LL (*ρ* = 0.57, *P* = 0.03) progression at final visit.

## Discussion

Clinically meaningful and highly responsive outcome measures are crucial for drug trials in CMT1A and other inherited neuropathies. Previous research including the 12‐month longitudinal data in this cohort has demonstrated quantitative lower limb muscle MRI to be a strong candidate, in particular showing cross‐sectional criterion validity through correlation with clinical measures,^9^, ^10^, ^12^, ^15^ and high responsiveness over 12 months.^12^, ^15^, ^16^, ^17^ The extended study reported here provides additional key evidence for the longitudinal validity for muscle MRI as a biomarker in CMT. The extended follow‐up duration to 4 years has allowed measurement of the rate of clinical progression using the CMT examination score, which shows strong correlation with rate of calf muscle fat fraction progression on MRI. Crucially, we have also demonstrated that the rate of progression in calf muscle fat fraction over 12 months correlates strongly with long‐term clinical progression up to 4 years. This provides support for calf muscle MRI to be used as a bridging biomarker in patients with CMT1A. In other words, in a clinical trial, a reduction in progression in calf muscle fat fraction at 1 year would be predicted to translate to long‐term slowing of disease progression based on clinical outcome measures. This is critical to allow clinical trials in CMT1A to demonstrate efficacy or lack of efficacy over a reasonable time frame, to allow candidate drugs to proceed to accelerated approvals, further studies or indeed be excluded as potentially effective interventions.

The second key outcome of this study is providing useful insights into optimal trial design. Specifically, as outcome measure responsiveness is a key determinant of study power, optimising responsiveness is crucial to allowing adequately powered studies in rare diseases. Responsiveness when represented as the SRM, which is the mean change divided by the sensitivity to change, affords two ways to increase its absolute value: increasing the mean change or reducing the standard deviation of the change. Of note, these data show that increasing study duration is not the most effective method to increase MRI outcome measure responsiveness in CMT1A with similar SRM seen in patients at 12 months, 27 months and 49 months (1.05, 1.05 and 0.95, respectively). The reason for this appears to be that calf muscle fat fraction progression is relatively consistent over 4 years in individual CMT1A patients. The effect of this is that although the mean change in muscle fat fraction increases with increased follow‐up interval, the standard deviation of the change also increases similarly—seen visually as the fanning out of data in Figure 3B. Progression in clinical measures such as the CMTES is more variable over any given 12‐month period. This is self‐evident as the CMTES is an integer scale with an average rate of change of 0.6 points per year in this study, broadly consistent with a natural history data of over 1100 patients over up to 6‐year follow‐up, where CMTES increased by 0.3 points per year.^3^ Conversely, calf muscle fat fraction is a continuous variable: the only limitation to measuring a small change is the reliability of the measurement, which has shown to be high.^23^ Hence, while increasing study duration increases responsiveness of clinical measures,^3^ the benefit of this approach is much smaller in quantitative MRI outcome measures.

We have previously demonstrated that baseline stratification increases MRI outcome measure responsiveness in CMT1A, by selection of patients with the greatest expected change in that measurement. Due to the length‐dependent pathology in CMT1A, in the youngest patients who are most mildly affected, there is no significant progression in calf fat fraction over 12 months, but progression is seen in foot muscles.^7^ Conversely in more severe CMT subtypes such as CMT2A, significant progression in thigh muscle fat fraction has been demonstrated.^19^ In the current study, even adults with normal baseline calf muscle fat fraction (in the control range <5%ff) show small but significant progression over 12 months (0.3 ± 0.3%ff) and 27 months (0.6 ± 0.4%ff) with large SRM due to the small standard deviations. Patients with intermediate fat fractions at baseline (5%–70%) have greater progression in muscle fat fraction at 12 months (2.0 ± 1.1) and 27 months (3.7 ± 2.3) with similar SRM as seen over 12 months in an independent US cohort.^15^ Approximately doubling SRM by selecting CMT1A patients for a clinical trial based on baseline calf muscle fat fraction will increase study power roughly fourfold versus an unselected cohort. In this study, we have also shown a very strong (*ρ* = 0.95, *P* < 0.001) correlation between mean calf Mercuri grade and quantitative calf muscle fat fraction. A mean modified Mercuri grade of calf muscles between 0.5 and 4 correlated in this study to an intermediate quantitative fat fraction. As T1w MRI scans can easily be performed, if included as an inclusion criteria, this would allow the screening MRI to be undertaken at patient identification sites and analysed rapidly. Alternatively, the CMTES, a simple clinical score, can also help with patient selection—a CMTES of less than 5 predicts normal calf muscle fat fraction with 80% accuracy, while a score greater than 7 predicts at least intermediate calf muscle fat fraction with 86% accuracy. These approaches should be confirmed and refined in cohorts of CMT1A patients.

Another approach to increase responsiveness is to select individual muscles rather than all muscles at the anatomical level. This has been an effective approach in facioscapulohumeral dystrophy where whole body muscle imaging was performed, and muscles of intermediate fat fraction selected as those to track longitudinally,^24^ and is being used as a secondary outcome measure in a phase 3 clinical trial (https://clinicaltrials.gov/study/NCT05397470). In the present study, we confirmed the findings of previous studies^6^, ^8^, ^9^, ^10^, ^11^ that of the calf muscles peroneus longus is most affected followed by tibialis anterior and medial gastrocnemius (Fig. 2A,B). However, the least severely affected patients had normal calf muscle fat fraction in all muscles, while the two most severely affected patents fat fraction greater than 70% in all muscles, so it is not unexpected that over the whole cohort average rate of progression is similar in all muscles. Indeed, due to the increased variability in fat fraction values from individual muscles, evidenced by the apparent asymmetry in progression of some muscles (Table 2), SRM at 12 months was generally lower in individual muscles than the overall measure, with only seven of 12 muscles demonstrating statistically significant change at 12 months. While the change at 27 months was significant in nine individual muscles, this approach does not improve outcome measure responsiveness in CMT1A. Another possible approach is to select muscles with baseline STIR hyperintensity; however, most patients in this study had no muscles with STIR hyperintensity, so this would not be a viable approach in CMT1A.

Rather, if trial design needed to include the full spectrum of disease severity, selection of a severity appropriate anatomical level for analysis should have greatest responsiveness. In children or young adults with normal calf fat fraction (<5%),^7^ foot muscle fat fraction would be selected; in patients of moderate severity with intermediate calf muscle fat fraction (5%–70%), calf muscle fat fraction would be selected, while in severely affected patients with calf fat fraction greater than 70%, thigh muscle fat fraction could be selected as the primary MRI outcome measure for that patient. This approach would need to be confirmed in a prospective study and should also be applicable to other length‐dependent neuropathies and we are utilising this approach in a current clinical trial of serine in hereditary sensory neuropathy type 1 (https://www.isrctn.com/ISRCTN17106427).

Although this study is the longest quantitative MRI natural history study to date, it has a number of limitations. The biggest limitation was that there was significant drop out over the course of the study such that only 10 patients remained at the final visit, which limited any subgroup analyses at this visit. Although the study benefitted from a single observer performing all the muscle segmentations for all subjects and visits, which validated the previous 12‐month data previously published^12^ which was segmented by a different observer, manual segmentation by a single observer may not be practical in large clinical trials and automated segmentations is an approach which is increasing in accuracy including in CMT1A.^16^, ^17^ Finally, the key measure of responsiveness of an outcome measure is to use it in a clinical trial. Lower limb muscle fat fraction is being utilised as secondary outcome measure in a current drug trial in SORD‐associated CMT (https://clinicaltrials.gov/study/NCT05397665) and as a primary outcome measure in a HSN1 (https://www.isrctn.com/ISRCTN17106427) which will Undoubtedly provide further valuable insights into MRI biomarkers in inherited neuropathies.

## Conclusions

CMT1A patients have progressive length‐dependent lower limb muscle fat accumulation, which has been shown previously by cross‐sectional studies to correlate with measures of strength, function and disease severity. It has large responsiveness over 12 months based on SRM measurement across three previous studies. In this study, we extended follow‐up to a mean duration of 49 months and demonstrated a constant rate of disease progression over this period, significant longitudinal correlation between CMTES and calf muscle fat fraction, and potential as a bridging biomarker by 1‐year change in fat fraction correlating with long‐term rate of clinical progression. Increasing study duration does not improve responsiveness; however, this can be increased by selecting patients with intermediate levels of fat fraction at baseline, or potentially considering a disease severity appropriate anatomical level for analysis. The value of MRI biomarkers in CMT should now be tested in clinical trials in CMT1A and is currently being tested in clinical trials in other forms of CMT.

## Author Contributions

MRBE, SS, TAY, MGH, JST, JMM and MMR contributed to conception and design of the study. MRBE, HAS, CDJS, SS, TAY and JMM contributed to acquisition and analysis of data. MRBE, HAS, JMM and MMR drafted a significant portion of the manuscript or figures.

## Conflicts of Interest

Nothing to report.

## Data Availability

The data that support the findings of this study are available from the corresponding author upon reasonable request.

## References

[1] Laurá M , Pipis M , Rossor AM , Reilly MM . Charcot‐Marie‐Tooth disease and related disorders: an evolving landscape. Curr Opin Neurol. 2019;32(5):641‐650. doi:10.1097/WCO.0000000000000735 31343428

[2] Pisciotta C , Pareyson D . Gene therapy and other novel treatment approaches for Charcot‐Marie‐Tooth disease. Neuromuscul Disord. 2023;33(8):627‐635. doi:10.1016/j.nmd.2023.07.001 37455204

[3] Fridman V , Sillau S , Acsadi G , et al. A longitudinal study of CMT1A using Rasch analysis based CMT neuropathy and examination scores. Neurology. 2020;94(9):e884‐e896. doi:10.1212/WNL.0000000000009035 32047073 PMC7238948

[4] Reilly MM , Herrmann DN , Pareyson D , et al. Trials for slowly progressive neurogenetic diseases need surrogate endpoints. Ann Neurol. 2023;93(5):906‐910. doi:10.1002/ana.26633 36891823 PMC10192108

[5] Burakiewicz J , Sinclair CDJ , Fischer D , Walter GA , Kan HE , Hollingsworth KG . Quantifying fat replacement of muscle by quantitative MRI in muscular dystrophy. J Neurol. 2017;264(10):2053‐2067. doi:10.1007/s00415-017-8547-3 28669118 PMC5617883

[6] Gallardo E , García A , Combarros O , Berciano J . Charcot‐Marie‐Tooth disease type 1A duplication: Spectrum of clinical and magnetic resonance imaging features in leg and foot muscles. Brain. 2006;129(2):426‐437. doi:10.1093/brain/awh693 16317020

[7] Doherty CM , Howard P , O'Donnell LF , et al. Quantitative foot muscle magnetic resonance imaging reliably measures disease progression in children and adolescents with Charcot–Marie–Tooth disease type 1A. Ann Neurol. 2024;96(1):170‐174. doi:10.1002/ana.26934 38613459

[8] Kim YJ , Kim HS , Lee JH , Yoon YC , Choi BO . Magnetic resonance imaging‐based lower limb muscle evaluation in Charcot‐Marie‐Tooth disease type 1A patients and its correlation with clinical data. Sci Rep. 2022;12(1):16622. doi:10.1038/s41598-022-21112-8 36198750 PMC9534835

[9] Bas J , Ogier AC , Le Troter A , et al. Fat fraction distribution in lower limb muscles of patients with CMT1A a quantitative MRI study. Neurology. 2020;94(14):e1480‐e1487. doi:10.1212/WNL.0000000000009013 31980579

[10] Sun X , Liu X , Zhao Q , Zhang L , Yuan H . Quantified fat fraction as biomarker assessing disease severity in rare Charcot–Marie–Tooth subtypes. Front Neurol. 2023;14:1334976. doi:10.3389/fneur.2023.1334976 38348112 PMC10859536

[11] Chung KW , Suh BC , Shy ME , et al. Different clinical and magnetic resonance imaging features between Charcot‐Marie‐Tooth disease type 1A and 2A. Neuromuscul Disord. 2008;18(8):610‐618. doi:10.1016/j.nmd.2008.05.012 18602827

[12] Morrow JM , Sinclair CDJ , Fischmann A , et al. MRI biomarker assessment of neuromuscular disease progression: a prospective observational cohort study. Lancet Neurol. 2016;15(1):65‐77. doi:10.1016/S1474-4422(15)00242-2 26549782 PMC4672173

[13] Cornett KMD , Wojciechowski E , Sman AD , et al. Magnetic resonance imaging of the anterior compartment of the lower leg is a biomarker for weakness, disability, and impaired gait in childhood Charcot–Marie–Tooth disease. Muscle Nerve. 2019;59(2):213‐217. doi:10.1002/mus.26352 30265406

[14] Bähr FS , Gess B , Müller M , et al. Semi‐automatic MRI muscle volumetry to diagnose and monitor hereditary and acquired polyneuropathies. Brain Sci. 2021;11(2):202. doi:10.3390/brainsci11020202 33562055 PMC7914808

[15] Morrow JM , Evans MRB , Grider T , et al. Validation of MRC Centre MRI calf muscle fat fraction protocol as an outcome measure in CMT1A. Neurology. 2018;91(12):e1125‐e1129. doi:10.1212/WNL.0000000000006214 30120135 PMC6161551

[16] O'Donnell LF , Pipis M , Thornton JS , et al. Quantitative MRI outcome measures in CMT1A using automated lower limb muscle segmentation. J Neurol Neurosurg Psychiatry. 2023;95:500‐503. doi:10.1136/jnnp-2023-332454 37979968

[17] Fortanier E , Hostin MA , Michel C , et al. One‐year longitudinal assessment of patients with CMT1A using quantitative MRI. Neurology. 2024;102(9):e209277. doi:10.1212/WNL.0000000000209277 38630962

[18] Kugathasan U , Evans MRB , Morrow JM , et al. Development of MRC Centre MRI calf muscle fat fraction protocol as a sensitive outcome measure in hereditary sensory neuropathy type 1. J Neurol Neurosurg Psychiatry. 2019;90:895‐906. doi:10.1136/jnnp-2018-320198 30995999

[19] Doherty CM , Morrow JM , Zuccarino R , et al. Lower limb muscle MRI fat fraction is a responsive outcome measure in CMT X1, 1B and 2A. Ann Clin Transl Neurol. 2024;11:607‐617. doi:10.1002/acn3.51979 38173284 PMC10963306

[20] Yushkevich PA , Piven J , Hazlett HC , et al. User‐guided 3D active contour segmentation of anatomical structures: significantly improved efficiency and reliability. NeuroImage. 2006;31(3):1116‐1128. doi:10.1016/j.neuroimage.2006.01.015 16545965

[21] Vivekanandam V , Seutterlin K , Matthews E , et al. Muscle MRI in periodic paralysis shows myopathy is common and correlates with intramuscular fat accumulation. Muscle Nerve. 2023;68(4):439‐450. doi:10.1002/mus.27947 37515374

[22] Mercuri E , Talim B , Moghadaszadeh B , et al. Clinical and imaging findings in six cases of congenital muscular dystrophy with rigid spine syndrome linked to chromosome 1p (RSMD1). Neuromuscul Disord. 2002;12(7‐8):631‐638. doi:10.1016/S0960-8966(02)00023-8 12207930

[23] Morrow JM , Sinclair CDJ , Fischmann A , et al. Reproducibility, and age, body‐weight and gender dependency of candidate skeletal muscle MRI outcome measures in healthy volunteers. Eur Radiol. 2014;24(7):1610‐1620. doi:10.1007/s00330-014-3145-6 24748539 PMC4046083

[24] Mellion ML , Widholm P , Karlsson M , et al. Quantitative muscle analysis in FSHD using whole‐body fat‐referenced MRI: composite scores for longitudinal and cross‐sectional analysis. Neurology. 2022;99(9):e877‐e889. doi:10.1212/WNL.0000000000200757 35750498

